# National and regional allergy and asthma programs: A comparative analysis and lessons for a German National Action Plan For Allergology 

**DOI:** 10.5414/ALX02666E

**Published:** 2026-11-05

**Authors:** Charlotte Köhler, Birgit Kalb, Sven Becker, Ludger Klimek, Lars Lange, Christian Vogelberg, Margitta Worm, Eckard Hamelmann

**Affiliations:** 1 Medical School OWL,; 2 Department of Pediatrics and Adolescent Medicine, Bethel Children’s Center, University Medical Center OWL, Bielefeld University, Bielefeld,; 3 Department of Otorhinolaryngology, Head and Neck Surgery, University Hospital Tübingen, Eberhard Karls University Tübingen, Tübingen,; 4 Allergy Center Wiesbaden, Wiesbaden,; 5 Department of Pediatrics and Adolescent Medicine, St. Marien Hospital, GFO Clinics, Bonn,; 6 Department of Pediatrics, University Hospital Carl Gustav Carus Dresden, TU Dresden, Dresden,; 7 Department of Dermatology, Venereology and Allergology, Charité Universitätsmedizin Berlin, Germany,; 8 German Society for Allergology and Clinical Immunology (DGAKI),; 9 German Academy of Allergology and Environmental Medicine (AeDA), and; 10 Society for Pediatric Allergology and Environmental Medicine (GPAU), Germany

**Keywords:** allergy, allergology, National Action Plan, health policy, implementation, integrated care

## Abstract

Background: Allergic diseases impose a substantial public health burden, yet Germany lacks a sustained national framework coordinating allergy-related care, prevention, education, research, and evaluation. We analyzed international allergy and asthma programs to identify principles relevant to a German National Action Plan (NAP) for Allergology. Materials and methods: A structured literature and policy review identified national and regional allergy and asthma initiatives through scientific databases, grey literature, and policy documents. Of 13 identified initiatives, 8 were included in the primary comparative analysis. Program characteristics and reported outcomes were descriptively assessed, and structural components were compared across nine analytical domains. Results: The programs differed in scope, implementation, governance, and evaluation. Recurring components included defined priorities, governance and accountability, multidisciplinary stakeholder involvement, integration of evidence-based care, professional and patient education, research and data infrastructure, monitoring, and sustainable resources. Several established programs reported favorable changes in healthcare utilization, disease management, patient quality of life, and healthcare costs. Predominantly observational evaluations, however, limited causal attribution. Eight interrelated strategic objectives were derived for consideration in the development of a German NAP. Conclusion: International programs cannot be directly transferred to Germany. Their recurring structural and implementation principles may nevertheless inform a coordinated national strategy linking unmet needs to measurable objectives, implementation responsibilities, resources, and prospective evaluation. The proposed framework requires further prioritization, operationalization, and stakeholder consensus.

## Introduction 

Allergic diseases are among the most common chronic non-communicable diseases and represent a substantial and persistent public health burden in Germany. More than 30% of adults and more than 20% of children are affected by at least one allergic disease, including allergic rhinitis, asthma, atopic dermatitis, food allergy, and other potentially severe allergic conditions [1]. Beyond their impact on physical and psychological well-being and quality of life, allergic diseases are associated with considerable healthcare utilization, loss of productivity, and socioeconomic costs [2]. Their future burden may be further influenced by environmental and climatic changes, including prolonged pollen seasons and altered allergen exposure [3]. 

Despite this high disease burden, allergy care in Germany remains fragmented across different medical specialties and sectors of care. Allergology is not established as an independent medical specialty but is provided by physicians from several disciplines with additional allergological qualification. Regional differences in specialist availability, insufficient reimbursement of allergological services, barriers to guideline-based diagnosis and treatment, and gaps in education and coordination have been identified as potential contributors to suboptimal care, particularly for patients with severe or complex allergic diseases [4, 5, 6, 7, 8, 9]. Previous initiatives have addressed several aspects of allergy prevention and care; however, Germany currently lacks a sustained comprehensive national framework that integrates prevention, diagnosis, treatment, education, research, and health policy within a common implementation and evaluation structure [10]. 

Clinical guidelines and individual improvement initiatives are essential for evidence-based care but cannot alone address the cross-sectoral and structural challenges associated with allergic diseases. Allergy prevention and management extend beyond specialist medical care and involve primary care, hospitals, pharmacies, schools and childcare facilities, workplaces, patient organizations, public health, environmental and food policy, research institutions, payers, and policymakers [10, 11, 12]. A National Action Plan (NAP) can provide an overarching framework to align these activities around shared priorities and translate them into coordinated measures with defined responsibilities, timelines, resources, and measurable outcomes. Rather than replacing existing guidelines or initiatives, such a framework may facilitate their integration into a coherent and sustainable national strategy [13, 14]. 

International experience provides an opportunity to inform the design of such a strategy. Over the past decades, several countries and regions have implemented asthma or allergy programs addressing different aspects of prevention, early diagnosis, disease management, professional and patient education, integrated care, research, and monitoring. Some long-term programs have been associated with favorable changes in healthcare utilization, disease management, quality of life, and costs, whereas more recent strategies provide contemporary models of governance, stakeholder involvement, and cross-sectoral coordination [15, 16, 17, 18, 19, 20, 21, 22, 23]. However, these initiatives differ substantially in disease scope, healthcare setting, implementation strategy, duration, and evaluation, limiting the direct transfer of individual interventions between healthcare systems. 

The aim of this review was therefore to comparatively analyze national and regional allergy and asthma action plans and programs in order to identify recurring structural components, implementation strategies, and lessons relevant to the development of a German National Action Plan for Allergology. Particular attention was given to priority setting, stakeholder involvement, care structures, education, implementation, governance, funding, and monitoring and evaluation. Based on these findings, we sought to derive principles and a proposed strategic framework for a German NAP that can be adapted to the specific structures and needs of the German healthcare system. 

## Materials and methods 

### Study design 

We conducted a structured literature and policy review with a comparative analysis of national and regional action plans, strategies, and implementation programs addressing allergic diseases and/or asthma. The objective was to identify recurring structural and implementation components that may inform the development of a NAP for Allergology in Germany. 

Given the heterogeneity of the identified initiatives with respect to geographic scope, disease focus, healthcare setting, duration, implementation approach, and evaluation design, the analysis was not intended to quantitatively compare program effectiveness. Instead, descriptive program characteristics and reported outcomes were extracted separately, while the comparative analysis focused on structural and implementation domains derived from the structured extraction and coding framework. 

### Search strategy and identification of programs 

A structured literature and policy search was conducted to identify published and publicly available national and regional allergy and asthma action plans, strategies, and implementation programs. PubMed/MEDLINE and the Cochrane Library were searched for scientific publications describing the development, implementation, or evaluation of such initiatives. Additional searches using BASE and targeted web searches were conducted to identify grey literature, governmental and policy documents, program reports, and publications from professional societies and relevant national or international organizations. 

Reference lists of relevant reviews, program reports, guidelines, and identified publications were additionally screened for eligible initiatives and accompanying implementation or evaluation studies. 

Search terms included combinations of terms related to allergic diseases and asthma (allergy, allergic disease, asthma) with terms describing organized health strategies and programs (*national action plan, national program/programme, national strategy, asthma program/programme, allergy program/programme, implementation program, disease management program*) and terms related to implementation and evaluation (*implementation, evaluation, outcome, healthcare utilization, cost, education, stakeholder*). 

Scientific publications were complemented by primary policy and program documents where these provided information on program objectives, governance, implementation structures, stakeholder participation, resources, or monitoring that was not available from peer-reviewed publications. 

The search was conducted between December 2025 and August 2026. Details of the search strategy, supplementary source assessment, and selection and classification of initiatives are provided in the Supplemental Methods and Supplemental Table S1. 

### Eligibility and classification of initiatives 

Initiatives were considered eligible if they:

- Addressed asthma, allergic diseases, or both;
- Represented an organized strategy or program extending beyond an individual clinical intervention or clinical guideline;
- Were implemented or designed at the national or regional healthcare-system level;
- Included defined objectives and at least one identifiable implementation component; and
- Provided sufficient published or publicly available information to characterize program structure, implementation, or evaluation.

Eligible initiatives were subsequently classified according to their role in the comparative analysis. Initiatives were included in the primary comparative analysis if sufficient documentation was available to characterize their program or strategy structure, implementation approach, and relevant components across the analytical domains. Documents that primarily represented recommendations, calls for action, clinical guidance, or policy proposals without a sufficiently developed implementation framework were retained as contextual sources but were not included as primary programs in the cross-program comparison. 

Purely clinical guidelines, individual local interventions without a broader implementation framework, and publications that did not provide sufficient information on program design or implementation were not considered as primary programs for comparative analysis. 

Because the identified initiatives differed substantially in scope and maturity, they were classified according to geographic level, disease scope, implementation period, principal strategic focus, and availability of evaluation data. Recently developed strategies for which outcome data were not yet available were included for analysis of contemporary strategy design, governance, and proposed implementation structures but were distinguished from implemented programs with available evaluation data. 

### Data extraction 

For each included initiative, information was extracted from program documents and associated publications using a structured extraction framework. Descriptive characteristics included country or region, program name, implementation period, geographic level, disease scope, principal strategic focus, and availability and type of evaluation data. These characteristics are summarized in Table 1. 

Reported program outcomes were extracted separately where available, including changes in healthcare utilization, disease control, morbidity, patient or professional knowledge, quality of life, sick leave or disability, medication use, and healthcare or societal costs. Outcomes were interpreted in relation to the design of the respective evaluation and were not treated as evidence of effectiveness of individual program components. 

### Comparative analytical framework 

Comparative analysis was conducted across 9 overarching analytical domains, corresponding to the framework presented in Table 2. These domains were informed by the structured extraction and coding framework detailed in the Supplemental Methods and Supplemental Table S3:

- **Priority setting and objectives:** Identification of disease burden and/or care gaps, definition of target populations and priorities, specification of program objectives, and translation of objectives into implementation activities.
- **Governance and accountability:** Coordinating organizations or networks, institutional or professional leadership, assignment of implementation responsibilities, and national or regional coordination.
- **Multidisciplinary and multisectoral stakeholder involvement:** Participation of primary care and specialist physicians, other healthcare professionals, professional societies, researchers, patients and patient organizations, healthcare institutions, governmental bodies, and other relevant societal actors.
- **Integration of evidence-based care into routine healthcare:** Approaches to early diagnosis, evidence-based treatment, structured follow-up, written management or action plans, decision support, referral structures, and coordination between primary and specialist care.
- **Professional education:** Continuing education, guideline dissemination, training of healthcare professionals, educational materials, conferences, and other knowledge- transfer activities.
- **Patient education and empowerment:** Patient and family education, self- management support, written action plans, communication strategies, public awareness, and health information.
- **Research, data, and surveillance:** Epidemiological data collection, research activities, registries or reporting systems, use of health-system data for monitoring, and development of data infrastructure.
- **Monitoring and evaluation:** Use of process and outcome indicators, repeated assessment, healthcare-utilization measures, clinical and patient-reported outcomes, and economic evaluation.
- **Resources and sustainability:** Organizational and financial resources, integration into existing healthcare structures, institutional or political support, and funding or resource considerations relevant to continued implementation.

A subject area was considered relevant when it was explicitly described as part of the respective program or strategy in the identified program documentation or associated publications. Presence of a subject area was used to characterize program structure and did not imply evidence of its independent effectiveness. The detailed coding scheme used for the comparative assessment, including the distinction between explicit focus, indirectly addressed, not mentioned, and not applicable, is described in the Supplemental Methods and Supplemental Table S3. 

### Comparative synthesis and derivation of lessons for Germany 

The 9 analytical domains were applied across the included initiatives to identify similarities and differences and to determine which structural and implementation components recurred despite differences in disease scope, healthcare system, geographic level, program duration, and evaluation design. 

Findings were synthesized narratively and comparatively. Because most program evaluations used observational designs, including longitudinal, survey-based, or pre-post approaches, and differed substantially in populations, endpoints, and follow-up periods, reported changes were interpreted as program-associated outcomes unless causal effects were supported by the respective study design. No quantitative pooling of outcomes was performed. 

Recurring subject areas identified across the programs were subsequently considered as potential transferable design and implementation principles for a German NAP Allergology. Transferability was defined as the potential applicability of an underlying principle within the German healthcare context rather than the direct adoption of an intervention, organizational model, or reported effect from another healthcare system. These principles informed the proposed strategic framework for a German NAP, while their prioritization, feasibility, and operationalization were considered to require subsequent assessment and stakeholder consensus within Germany. 

## Results 

### Characteristics of the identified programs 

The structured search and supplementary source assessment identified 13 potentially relevant initiatives, including national or regional programs, strategies, policy initiatives, and guideline-based approaches. Eight initiatives met the criteria for inclusion in the primary comparative analysis: the Australian National Asthma Campaign, the Finnish Asthma Programme, the Finnish Allergy Programme, the Towards Excellence in Asthma Management (TEAM) programme (from Canada), the Program for Control of Asthma and Allergic Rhinitis (ProAR) (from Brazil), the Primary Care Asthma Pilot Project (PCAPP) (from Canada), the Polish National Programme of Early Diagnosis and Therapy of Asthma (POLASTMA), and the UK National Allergy Strategy. The remaining initiatives were retained as contextual sources where relevant to the historical or policy background but were not included in the primary cross-program comparison. Details of the identification, classification, and selection of initiatives are provided in the Supplemental Methods and Supplemental Table S1. The 8 included initiatives differed substantially in geographic scope, disease focus, duration, implementation approach, and availability of evaluation data (Table 1). The initiatives included national programs with long-term implementation and follow-up, regional disease-management and primary-care implementation programs, and a recently developed comprehensive national allergy strategy. 

Most earlier initiatives focused on asthma, including the Australian National Asthma Campaign (NAC), the Finnish Asthma Programme, the Towards Excellence in Asthma Management programme (TEAM), the Primary Care Asthma Pilot Project (PCAPP), and the Polish National Programme of Early Diagnosis and Therapy of Asthma (POLASTMA). ProAR in Salvador addressed asthma and allergic rhinitis, whereas the Finnish Allergy Programme and the UK National Allergy Strategy covered allergic diseases more broadly. The Finnish Asthma Programme (1994 – 2004) and Finnish Allergy Programme (2008 – 2018) were analyzed separately because they differed in disease scope, objectives, and implementation period [15, 16, 24, 25, 26]. 

The maturity of the initiatives and the available evidence also differed. Established programs were accompanied predominantly by observational, longitudinal, survey-based, or pre-post evaluations assessing healthcare utilization, disease management, knowledge, quality of life, or economic outcomes [15, 16, 17, 19, 20, 21, 22, 27, 28, 29, 30, 31, 32]. In contrast, the recently developed UK National Allergy Strategy was included to assess contemporary strategy design, governance, and proposed implementation structures; long-term outcome data are not yet available [23]. 

### Program-specific findings 

**Australian National Asthma Campaign**

The Australian National Asthma Campaign, launched in 1990, combined public education and mass-media activities with professional education and promotion of structured asthma management [28, 33]. Evaluations reported increased asthma-related knowledge and use of management plans as well as changes in diagnosis, referral patterns, and healthcare utilization. Reductions in hospital readmissions were observed in some age groups, although findings were not uniform across populations [27, 28, 29]. 

**Finnish Asthma Programme and Finnish Allergy Programme**

The Finnish Asthma Programme (1994 – 2004) combined early diagnosis and active treatment with guided self-management, professional education, evidence-based recommendations, and coordination between primary and specialist care [15, 24]. During the program period, reductions in hospital use, disability, and costs were reported despite an increasing number of patients receiving treatment [15]. 

The subsequent Finnish Allergy Programme (2008 – 2018) broadened the scope to allergic diseases and combined prevention and disease management with professional and patient education and a revised approach to allergen avoidance and tolerance. Implementation involved healthcare institutions, governmental bodies, professional and patient organizations, pharmacies, and other societal actors and was accompanied by defined goals and longitudinal monitoring [16, 25, 26]. Favorable trends were reported for several indicators, including healthcare utilization, occupational allergy, sick leave, and costs [16, 26]. 

Both programs were evaluated predominantly using population-level observational and temporal data; reported changes were therefore considered program-associated developments rather than evidence of uniform causal effects. 

**Towards Excellence in Asthma Management**

The Canadian TEAM initiative (1999 – 2007) used a population-based disease-management approach to address gaps between evidence-based recommendations and routine asthma care. Its components included identification of regional care gaps, professional education, patient self- management, improved patient–physician communication, and primary-care management approaches [17, 18]. During the eight-year program, improvements in asthma-related knowledge, management, and quality of life and reductions in selected measures of healthcare utilization and sick days were reported. 

**Program for Control of Asthma and Allergic Rhinitis**

ProAR in Salvador, Brazil (2003 – 2006), combined multidisciplinary guideline-based care with patient education, regular follow-up, and access to medication [19]. Following implementation, reductions in emergency visits, hospitalizations, and oral corticosteroid use were reported, together with improvements in asthma control and quality of life in evaluated subgroups [19, 21, 22]. Economic analyses also reported reductions in individual and societal costs [31, 32]. 

**Primary Care Asthma Pilot Project**

The Canadian PCAPP (2003 – 2006) implemented an evidence-based asthma-care model in primary care using a community-based participatory approach [30]. The intervention combined professional education, written action plans, self-management and decision support, structured patient contacts, and designated asthma coordinators. In a longitudinal pre-post evaluation, reductions in uncontrolled symptoms, exacerbations, emergency healthcare use, and sick days were reported 1 year after implementation [30]. 

**POLASTMA**

POLASTMA (2008 – 2014) aimed to improve asthma diagnosis, treatment, and disease management in Poland through evidence-based recommendations, professional education, and organizational measures, with particular emphasis on strengthening routine asthma care in primary and pediatric care. Program activities included educational materials, professional training, reference-center structures, conferences, e-health approaches, and cooperation with patient organizations and healthcare stakeholders. The available evidence primarily describes program activities and implementation rather than clinical or healthcare outcomes [34]. 

**UK National Allergy Strategy**

The UK National Allergy Strategy (2025 – 2035) differs from the predominantly asthma-focused programs in its broad allergy-specific and cross-sectoral scope. It addresses prevention, diagnosis and treatment, patient safety, workforce development, education, data, research, and coordination and involves clinicians, patients, researchers, and other stakeholders [23]. Proposed structural elements include strengthened primary care, improved allergy-related data collection and surveillance, workforce planning, school-related measures, patient involvement, accountability, monitoring, and sustainable funding [23]. 

Because the strategy is recent, long-term outcome data are not yet available. The strategy was therefore included as an example of contemporary strategy design and proposed governance and implementation structures, not as evidence of effectiveness. 

### Cross-program comparative findings 

Despite substantial differences in disease scope, healthcare setting, geographic level, duration, and evaluation design, comparison of the initiatives identified recurring structural and implementation components (Table 2). These comprised priority setting and defined objectives; governance and accountability; multidisciplinary and multisectoral stakeholder involvement; integration of evidence-based care into routine healthcare; professional education; patient education and empowerment; research, data and surveillance; monitoring and evaluation; and resources and sustainability. 

### Priority setting and defined objectives 

The programs generally linked identifiable problems in disease management, healthcare utilization, access to care, professional knowledge, or patient self-management to defined objectives and implementation activities. The Finnish programs combined national objectives with corresponding measures and longitudinal monitoring [15, 16, 24, 25, 26]. TEAM and PCAPP focused on gaps between evidence-based recommendations and routine care [17, 18, 30], whereas POLASTMA emphasized early diagnosis, professional education, and organization of asthma management [34]. 

### Governance, accountability, and stakeholder involvement 

Multidisciplinary stakeholder involvement was a recurring feature, although governance structures differed substantially. Participating groups included primary care physicians, specialists, nurses, pharmacists, professional societies, researchers, patient organizations, healthcare institutions, health authorities, insurers, and governmental bodies [15, 16, 17, 18, 23, 24, 25, 26, 30, 34]. Governance ranged from national coordination, as in Finland, to regional healthcare networks and program-specific structures, as in TEAM and PCAPP, while the UK strategy proposes a broader cross-sectoral framework [15, 16, 17, 18, 23, 24, 25, 26, 30]. 

### Integration of evidence-based care into routine healthcare 

Several programs sought to translate evidence-based recommendations into routine care through combinations of early diagnosis, structured follow-up, written action plans, decision support, referral structures, multidisciplinary care, and coordination between primary and specialist care [18, 19, 23, 24, 30, 33, 34]. Primary care was particularly prominent in the Finnish Asthma Programme, TEAM, PCAPP, and POLASTMA [18, 24, 30, 34], whereas ProAR additionally combined guideline-based management with medication access and continued follow-up [19]. 

### Professional and patient education 

Professional interventions included continuing education, guideline dissemination, training, educational materials, and other forms of knowledge transfer [18, 19, 23, 24, 25, 30, 33, 34]. Patient-oriented approaches included structured education, self-management support, written action plans, family involvement, public information, and awareness activities [18, 19, 23, 24, 25, 28, 30, 33, 34]. Because these measures were generally embedded within multicomponent programs, their independent contribution to reported outcomes could not be determined. 

### Research, data, and surveillance 

Research and data-related components varied between initiatives and included epidemiological assessment, program-related research, structured data collection, reporting systems, and use of healthcare data for monitoring [15, 16, 17, 18, 23, 24, 25, 26, 34]. These components were particularly prominent in the Finnish programs and are also incorporated into the proposed infrastructure of the UK strategy [15, 16, 23, 24, 25, 26]. 

### Monitoring and evaluation 

Evaluation approaches differed considerably between programs. Reported indicators included hospitalizations, emergency visits, disease control, disability or sick leave, knowledge, quality of life, medication use, and healthcare or societal costs [15, 16, 17, 19, 21, 22, 27, 28, 29, 30, 31, 32]. The Finnish programs provided the longest periods of follow-up, whereas other initiatives used survey-based, longitudinal, or pre-post approaches [15, 16, 17, 19, 21, 22, 27, 28, 29, 30]. Heterogeneity in populations, endpoints, study designs, and follow-up periods precluded direct quantitative comparison of program effects. 

### Resources and sustainability 

Implementation required organizational and financial resources for coordination, education, communication, treatment access, data collection, and evaluation. Several initiatives involved governmental bodies, healthcare organizations, professional societies, insurers, or other institutional partners [18, 19, 23, 24, 25, 26, 34]. However, the extent to which funding mechanisms and long-term sustainability were explicitly reported varied between programs. Long-term national implementation was most clearly documented for the Finnish programs, whereas the UK strategy explicitly incorporates leadership, accountability, and sustainable funding within its proposed framework [15, 16, 23, 24, 25, 26]. 

### Overall synthesis 

No single intervention was consistently present across all initiatives or could be isolated as the principal explanation for reported outcomes. Rather, the recurring characteristic of the more comprehensive programs was the coordination of multiple clinical, educational, organizational, and data-related components through defined objectives, stakeholder involvement, implementation mechanisms, and monitoring. 

## Discussion 

### Principal findings and lessons from existing programs 

This comparative analysis identified substantial heterogeneity among national and regional asthma and allergy programs with regard to disease scope, healthcare setting, duration, governance, implementation, and evaluation. Nevertheless, recurring structural components were evident across programs, including evidence-based priority setting, defined objectives, multidisciplinary stakeholder involvement, integration of evidence-based interventions into routine care, professional and patient education, data collection, monitoring and evaluation, and organizational structures supporting implementation. 

The principal finding is therefore not the superiority of a particular intervention or healthcare model. Rather, the reviewed programs demonstrate different approaches to coordinating multiple complementary interventions within a common framework and linking strategic objectives to implementation and evaluation. The Finnish Asthma Programme and the subsequent Finnish Allergy Programme provide the most comprehensive long-term examples identified in this review [15, 16, 24, 25, 26]. TEAM, ProAR, and PCAPP demonstrate how coordinated approaches can also be implemented at the regional or primary-care level [17, 18, 19, 30], whereas POLASTMA illustrates a professional-society-led approach combining early diagnosis, education, and organizational development [34]. The recently developed UK National Allergy Strategy extends this concept towards a broader cross-sectoral allergy strategy, although its recent development and the absence of long-term outcome data preclude conclusions regarding effectiveness [23]. 

These differences are important for interpretation. Neither individual interventions nor reported effect sizes can be transferred directly between healthcare systems. Furthermore, most available evaluations were observational or based on temporal or pre-post comparisons. What appears more transferable are the underlying design and implementation principles: identification of relevant care gaps, prioritization of objectives, involvement of those responsible for and affected by implementation, integration into existing care structures, defined responsibilities, and prospective monitoring. 

### From international experience to unmet needs in Germany 

Germany has previously undertaken several allergy-related policy and professional initiatives. The federal “Aktionsplan gegen Allergien” (2007 – 2012) addressed public outreach campaigns, prevention measures, and actions to improve cooperation between affected individuals, policymakers, and other stakeholders [35, 36]. German allergological societies subsequently called for a National Action Plan Allergy, proposing action fields including prevention, education, continuing professional development, timely medical care, and evaluation [10]. In 2020, a further political initiative emphasized prevention, information, evidence-based care, research, and monitoring [11]. Together, these initiatives demonstrate that the need for coordinated national action in allergology has been recognized repeatedly in Germany. 

However, Germany currently lacks a sustained comprehensive national framework that integrates allergy-related prevention, healthcare, education, research, and monitoring through jointly defined priorities, responsibilities, implementation mechanisms, and measurable objectives. Importantly, the available evidence allows us to establish the sequence of previous initiatives, but not to determine conclusively why they did not result in such a sustained framework. 

This coordination challenge is particularly relevant to allergology. In Germany, allergological care is distributed across several medical specialties and levels of care rather than organized within an independent medical specialty. Prevention, diagnosis, treatment, and long-term management therefore depend on interfaces between primary care, pediatrics, dermatology, otorhinolaryngology, pneumology, specialist allergology, hospitals, and other healthcare professions. Depending on disease and setting, additional interfaces include pharmacies, schools and childcare, workplaces and occupational health, patient organizations, public health, environmental policy, and food-related regulation. 

Accordingly, relevant unmet needs are not exclusively clinical. They also concern coordination and continuity of care, access to allergological expertise, availability of diagnostic and therapeutic allergens, availability of approved pediatric medication, professional competencies, patient empowerment, data availability, implementation, and governance. The available German literature and previous initiatives describe deficits in several of these areas, including fragmented care structures and access to allergological expertise [5, 6, 7, 8, 9], limitations in professional education and competencies [4, 5], gaps in guideline-based diagnosis and care [5, 7, 9], and the need for improved monitoring [10, 11]. A German NAP would therefore add value only if it addresses these interfaces and structural gaps rather than duplicating existing clinical guidelines, professional recommendations, or individual initiatives. 

### Implications for the design of a German NAP Allergology 

The international comparison identifies several principles that may distinguish a future NAP from isolated or time-limited initiatives. 

First, priority setting should start with systematically identified unmet needs. Disease burden, care gaps, inequalities in access, patient perspectives, and implementation barriers should form the basis for selecting priority areas. This approach would support prioritization according to relevance and feasibility rather than the development of an unrestricted catalogue of desirable activities. 

Second, priorities should be translated into measurable implementation targets. Based on the recurring features identified across programs, each strategic objective of a future German NAP should be linked to defined measures, responsible actors, realistic timelines, and indicators. The reviewed programs used heterogeneous outcomes, including healthcare utilization, disease control, knowledge, quality of life, sick leave, and costs [15, 16, 17, 19, 21, 22, 27, 28, 29, 30, 31, 32]. For a German NAP, a limited set of process and outcome indicators should therefore be defined before implementation and supported by baseline data wherever possible. 

Third, implementation should be designed concurrently with strategy development. Existing healthcare structures, clinical guidelines, professional networks, educational structures, registries, and data sources should be used wherever appropriate. New structures should primarily address gaps that cannot be covered through existing mechanisms. This may improve feasibility and reduce duplication. 

Fourth, stakeholder participation needs to be complemented by governance. Patients and patient organizations, relevant medical specialties, primary care, other healthcare professions, researchers, payers, policymakers, and representatives of relevant non- healthcare sectors should contribute to priority setting and implementation. However, consensus alone does not ensure implementation. Priorities require identifiable leadership, assigned responsibilities, decision-making structures, and accountability. 

Fifth, monitoring and evaluation should be integral to the NAP from its inception. Process indicators are required to assess whether agreed measures are implemented, while outcome indicators are needed to determine whether relevant changes in care and patient outcomes occur. Long-term monitoring was particularly prominent in the Finnish programs [15, 16, 25, 26], whereas the heterogeneity of evaluation approaches in other programs illustrates the limitations of retrospective assessment. 

Finally, sustainability should be addressed explicitly during program design. Coordination, education, data infrastructure, research, communication, and evaluation require organizational capacity and resources. Political commitment, clearly assigned responsibility, and sustainable financing should therefore be regarded as implementation requirements rather than additions to an otherwise completed action plan. 

Based on the recurring domains identified across the reviewed programs and their relevance to the German setting, 8 interrelated strategic objectives are proposed for consideration in the subsequent stakeholder consultation and consensus-building process (Figure 1). These encompass prevention and early detection; equitable access to evidence-based diagnosis and treatment; coordinated care pathways; patient empowerment and health literacy; professional education and allergological competencies; research, data and surveillance; monitoring and evaluation; and governance with sustainable resources. 

These objectives should not be interpreted as a finalized or formally agreed German NAP. They constitute a proposed framework derived from the present comparative analysis. Their prioritization and translation into specific measures, responsibilities, timelines, and measurable targets require systematic assessment within the German healthcare system and structured stakeholder consensus. 

Importantly, the proposed strategic objectives shown in Figure 1 represent fields of action and mechanisms for change, whereas reduced avoidable morbidity and complications, improved quality of life and patient safety, greater equity of care, and more efficient use of healthcare resources are intended long-term outcomes. These outcomes should be prospectively evaluated rather than assumed as consequences of implementing a NAP. 

### From strategy to implementation 

The comparison also highlights what should distinguish a future German NAP from another collection of recommendations. Its added value would lie in providing an implementation architecture that connects evidence, priorities, consensus, implementation, and evaluation. 

For each priority area, the development process should therefore address five fundamental questions: What is the unmet need? What measurable change is intended? Which measures are required? Who is responsible for implementation? How and when will progress be evaluated? 

Such an approach would link strategic objectives to accountability and allow measures to be adapted when monitoring demonstrates insufficient implementation or effectiveness. It would also provide a framework for continuity beyond individual projects or policy cycles. 

The potential value of a national action plan therefore lies not primarily in introducing individual interventions, but in coordinating evidence-based interventions across sectors within a common framework of priorities, responsibilities, timelines, resources, and measurable outcomes. 

### Limitations 

Several limitations need to be considered. The identified programs were heterogeneous with regard to disease scope, geographic level, healthcare system, target population, duration, and implementation context. Most available evaluations used observational designs, including longitudinal, survey-based, or pre-post approaches, limiting causal attribution of reported changes to the respective programs. Endpoints and follow-up periods differed substantially, precluding quantitative comparison or meta-analysis. 

The evidence base may additionally be affected by publication and reporting bias, since well- documented or favorable program experiences may be more likely to be published. Relevant information was partly obtained from grey literature and policy documents with variable reporting standards. Most implementation evidence identified in this review derives from asthma programs; transfer of these principles to the broader and more heterogeneous field of allergology therefore requires empirical validation within the German NAP development process. Conversely, the recent UK National Allergy Strategy provides a contemporary allergy- specific model but does not yet provide evidence of effectiveness [23]. 

As this was a structured literature and policy review rather than a systematic review, the search may not have identified all relevant initiatives. In addition, no formal risk-of-bias assessment across the heterogeneous program evaluations and policy documents was performed. 

Finally, transferability between healthcare systems is inherently limited. The present analysis therefore identifies recurring structural and implementation principles rather than interventions that can be directly adopted in Germany. Their relevance, feasibility, prioritization, and operationalization need to be established within the German healthcare context. 

## Conclusion 

The comparative analysis of national and regional asthma and allergy programs identified recurring structural and implementation principles relevant to the development of a German National Action Plan for Allergology. Despite substantial differences in healthcare systems, program scope, and evaluation, these initiatives illustrate how multiple complementary components can be coordinated within an overarching framework linking priorities, implementation, and evaluation. 

For Germany, the added value of a National Action Plan would not lie in creating another catalogue of recommendations, but in providing a framework that translates identified unmet needs into prioritized and measurable objectives, defined responsibilities, implementation mechanisms, and prospective evaluation. 

International experience cannot be transferred directly to the German healthcare system. However, the principles identified in this review can inform the structured development of a German NAP Allergology and the subsequent stakeholder consensus-building process. Integrating implementation, governance, resources, and evaluation from the outset will be essential to establish a sustainable and evaluable national strategy and to determine prospectively whether it improves allergy care and patient outcomes. 

## Ethics approval and consent to participate 

None. 

## Data availability 

All data analyzed in this review were obtained from publicly available publications and policy documents cited in the article and its Supplementary Material. The structured comparative coding is provided in Supplementary Table S3. 

## Use of artificial intelligence 

Artificial intelligence (AI) tools were not used in the preparation of this manuscript. 

## Funding 

This work was supported by the German Society for Allergology and Clinical Immunology (DGAKI), the German Society for Pediatric Allergology and Environmental Medicine (GPAU), and the German Academy of Allergology, Immunology and Environmental Medicine (AeDA), which provided equal financial support to CK. The other authors received no specific funding for this work. 

## Conflict of interest 

### Financial interests 

EH received grants from the Federal Ministry of Health (BMG) and the Federal Ministry for Research, Technology, and Space (BMFTR) and the Innovation Fonds of the Federal Joint Committee (G-BA). He received honorarium for lectures or advisory boards from ALK, Almirall, AstraZeneca, DBV, Leti, Sanofi. 

LK has received research grants from Allergy Therapeutics/Bencard, United Kingdom/Germany; ALK-Abelló, Denmark; Allergopharma, Germany; Aimmune, U.S.; ASIT Biotech, Belgium; AstraZeneca, Sweden; Bionorica, Germany; BioNTech, Germany; Biomay, Austria; Blueprint, U.S.; Boehringer Ingelheim, Germany; Celltrion, South Korea; Circassia, USA; Chiesi, Italy; Cytos, Switzerland; Curalogic, Denmark; HAL, the Netherlands; Lofarma, Italy; Menarini, Italy; Viatris/Mylan, USA; Novartis, Switzerland; Leti, Spain; ROXALL, Germany; GlaxoSmithKline (GSK), United Kingdom; Sanofi, France; Stallergenes, France; Thermo Fisher, USA; and/or served as a speaker or consultant for the pharmaceutical companies listed above. 

CV received grants from German Research Foundation and Boehringer Ingelheim and received honorarium for lectures or advisory boards from ALK Abelló, Allergopharma, AstraZeneca, Bencard Allergie, Celltrion, Orion Pharma, Sanofi Aventis, Stallergenes. 

BK received grants from the Federal Ministry of Health (BMG) and the German Research Foundation (DFG). She received honorarium for advisory boards and lectures from ALK and Viatris. 

MW declares the receipt of honoraria or consultation fees within the last 3 years by the following companies: AbbVie Deutschland GmbH & Co. KG, ALK-Abelló Arzneimittel GmbH, Allergopharma GmbH & Co KG, Almirall Hermal GmbH, Amgen GmbH, AstraZenceca GmbH, Bayer AG, Bencard Allergy GmbH, Bioprojet Pharma, Boehringer Ingelheim Pharma GmbH &Co.KG, Bristol Myers Squibb GmbH & Co. KGaA, Lilly Deutschland GmbH, Galderma Laboratorium GmbH, Infectopharm Arzneimittel und Consilium GmbH, LEO Pharma GmbH, Mylan Germany GmbH (A Viatris Company), Octapharm AG, Pfizer Pharma GmbH, Sanofi-Aventis Deutschland GmbH/Genzyme Europe B. B., Stallergenes GmbH, and HAL Allergy. 

### Non-financial/professional interests 

EH is the President of the DGAKI and President of the German Asthma Net (GAN). MW is the Past-President of the DGAKI. LL is the President, CV the Past-President of the GPAU. LK is currently president of the AeDA, a member of the board of the Governance Committee of the European Academy of Allergology and Clinical Immunology (EAACI), and editor-in-chief of AllergoJournal and AllergoJournal International. SB is member of the Board of Directors of the AeDA. CK and BK declare no conflicts of interest. 

## Authors’ contributions 

CK: Methodology; investigation; data curation; formal analysis; writing – original draft. BK: Validation of coding; writing – review & editing. 

SB, LK, LL, CV, MW: Writing – review & editing. 

EH: Conceptualization; writing – original draft; writing – review & editing; supervision; project administration. 

**Table 1.** Characteristics of national and regional allergy and asthma programs included in the comparative analysis.

**Country/region**	**Program**	**Period**	**Level**	**Disease scope**	**Main strategic focus**	**Evaluation status**
Australia	National Asthma Campaign (NAC)	from 1990	National	Asthma	Public awareness; professional education; structured asthma management	Observational and survey-based evaluations available [27, 28, 29, 33]
Finland	Finnish Asthma Programme	1994 – 2004	National	Asthma	Early diagnosis and treatment; guided self-management; professional education; integration of primary and specialist care	Long-term observational evaluation available [15, 24]
Finland	Finnish Allergy Programme	2008 – 2018	National	Allergic diseases	Prevention; improved allergy management; education; promotion of tolerance; broader societal involvement	Long-term implementation and outcome evaluation available [16, 25, 26]
Canada/Quebec	Towards Excellence in Asthma Management (TEAM)	1999 – 2007*	Regional	Asthma	Reduction of evidence-to- practice gaps; professional and patient education; self- management; primary care	Program and outcome evaluations available [17, 18]
Brazil/Salvador	Program for Control of Asthma and Allergic Rhinitis (ProAR)	2003 – 2006, 2006 only interim results	Regional	Asthma and allergic rhinitis	Access to guideline-based treatment; medication access; patient education; multidisciplinary care	Clinical, healthcare-utilization, and economic evaluations available [19, 20, 21, 22, 31, 32]
Canada	Primary Care Asthma Pilot Project (PCAPP)	2003 – 2006	Regional/ community-based	Asthma	Implementation of evidence- based asthma care in primary care; self-management; decision support; coordination	Longitudinal pre-post evaluation available [30]
Poland	National Programme of Early Diagnosis and Therapy of Asthma (POLASTMA)	2008 – 2014	National	Asthma	Early diagnosis; professional education; treatment and disease management; research; primary-care capacity	Program description and educational/ implementation reports available [34, 37]
United Kingdom	National Allergy Strategy 2025 – 2035	2025 – 2035	National	Allergic diseases	Cross-sectoral strategy addressing prevention, care, patient safety, workforce, data, research, and policy	Strategy/early implementation phase; outcome evaluation not yet available [23]

NAC = National Asthma Campaign; TEAM = Towards Excellence in Asthma Management; ProAR = Program for Control of Asthma and Allergic Rhinitis; PCAPP = Primary Care Asthma Pilot Project. *The final report describes an eight-year research cycle ending with the second intervention series in 2007; individual preparatory activities had begun earlier.

**Table 2.** Recurring structural and implementation components identified across the reviewed programs.

**Analytical domain**	**Recurring components identified across programs**	**Programs in which components were identified**
Priority setting and objectives	Identification of disease burden and/or care gaps; definition of target populations; specified program objectives; translation of objectives into implementation activities	Finnish Asthma Programme [15, 24]; Finnish Allergy Programme [16, 25, 26]; TEAM [17, 18]; ProAR [19]; PCAPP [30]; POLASTMA [34]; UK National Allergy Strategy [23]
Governance and accountability	Coordinating organizations or networks; institutional or professional leadership; assignment of implementation responsibilities; national or regional coordination	Finnish Asthma Programme [15, 24]; Finnish Allergy Programme [16, 25, 26]; TEAM [17, 18]; PCAPP [30]; POLASTMA [34]; UK National Allergy Strategy [23]
Multidisciplinary and multisectoral stakeholder involvement	Participation of primary care and specialist physicians, other healthcare professionals, professional societies, researchers, patients, patient organizations, healthcare institutions and, depending on program scope, governmental and other societal actors	Australian NAC [33]; Finnish Asthma Programme [15, 24]; Finnish Allergy Programme [16, 25, 26]; TEAM [17, 18]; ProAR [19]; PCAPP [30]; POLASTMA [34]; UK National Allergy Strategy [23]
Integration of evidence- based care into routine healthcare	Early diagnosis; evidence-based treatment; structured follow-up; written management/action plans; decision support; improved referral structures; coordination between primary and specialist care	Australian NAC [33]; Finnish Asthma Programme [24]; TEAM [18]; ProAR [19]; PCAPP [30]; POLASTMA [34]; UK National Allergy Strategy [23]
Professional education	Continuing education; guideline dissemination; training of primary care physicians and specialists; educational materials; conferences and other knowledge-transfer activities	Australian NAC [33]; Finnish Asthma Programme [24]; Finnish Allergy Programme [25]; TEAM [18]; ProAR [19]; PCAPP [30]; POLASTMA [34]; UK National Allergy Strategy [23]
Patient education and empowerment	Patient and family education; self-management support; written action plans; communication strategies; public awareness and health information	Australian NAC [28, 33]; Finnish Asthma Programme [24]; Finnish Allergy Programme [25]; TEAM [18]; ProAR [19]; PCAPP [30]; POLASTMA [34]; UK National Allergy Strategy [23]
Research, data, and surveillance	Epidemiological data collection; research activities; registries/reporting systems; use of health-system data for monitoring; development of data infrastructure	Finnish Asthma Programme [15, 24]; Finnish Allergy Programme [16, 25, 26]; TEAM [17, 18]; POLASTMA [34]; UK National Allergy Strategy [23]
Monitoring and evaluation	Process and outcome indicators; repeated assessment; healthcare-utilization measures; clinical and patient-reported outcomes; economic evaluation	Australian NAC [27, 28, 29]; Finnish Asthma Programme [15]; Finnish Allergy Programme [16, 26]; TEAM [17]; ProAR [19, 21, 22, 31, 38]; PCAPP [30]
Resources and sustainability	Organizational resources; integration into existing healthcare structures; institutional or political support; funding or resource considerations for continued implementation	Finnish Asthma Programme [24]; Finnish Allergy Programme [25, 26]; TEAM [18]; ProAR [19]; POLASTMA [34]; UK National Allergy Strategy [23]

NAC = National Asthma Campaign; TEAM = Towards Excellence in Asthma Management; ProAR = Program for Control of Asthma and Allergic Rhinitis; PCAPP = Primary Care Asthma Pilot Project. Presence of a component indicates that it was described as part of the respective program or strategy; it does not imply evidence of effectiveness of that component. Reported program-specific outcomes and evaluation measures are presented in Supplemental Table S3.

**Figure 1.**
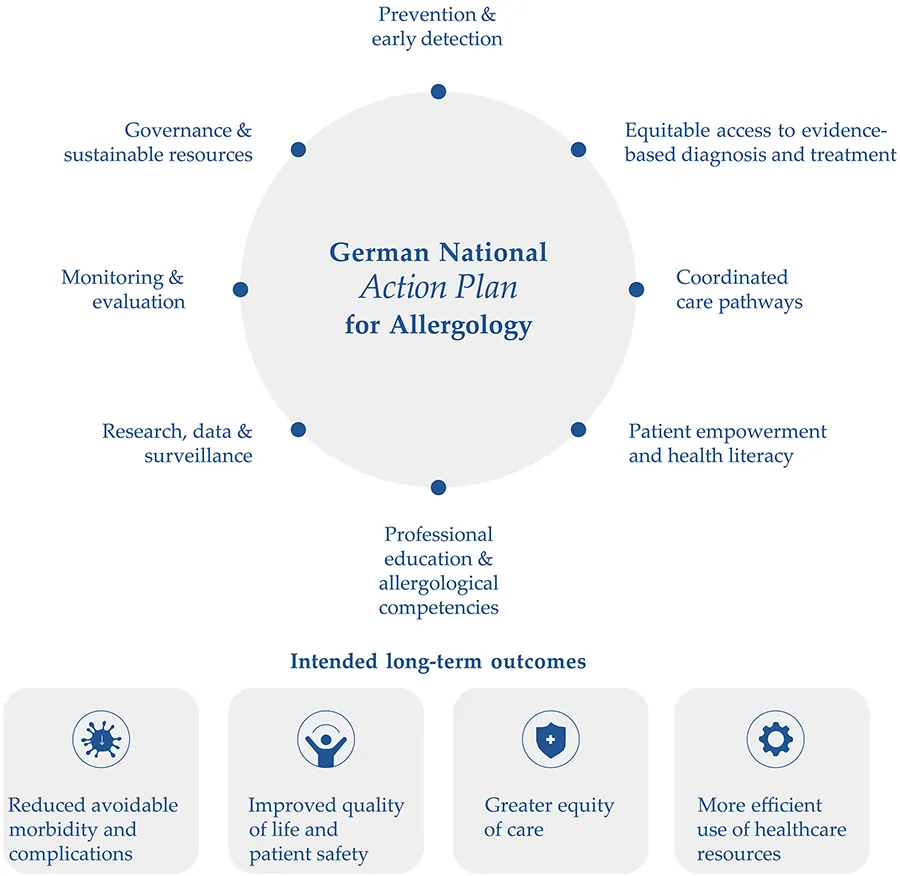
Proposed strategic objectives for a German National Action Plan for Allergology. Eight interrelated strategic objectives derived from the comparative analysis of national and regional allergy and asthma programs are proposed for consideration in the development of a German National Action Plan for Allergology. The proposed objectives represent a conceptual framework and require further prioritization, operationalization, and stakeholder consensus within the German healthcare context. Intended long-term outcomes include reduced avoidable morbidity and complications, improved quality of life and patient safety, greater equity of care, and more efficient use of healthcare resources.

### Supplemental material

Supplemental material Supplemental material
