# Supplementary material for: National and regional allergy and asthma programs: A comparative analysis and lessons for a German National Action Plan For Allergology
Source: Allergol Select. 2026 Nov 5;10:385–402. doi: 10.5414/ALX02666E (PMC13750063; doi:10.5414/ALX02666E)
Supplement: Supplementary file 1 [file allergologieselect-10-385-S01.pdf]

Supplement

Search strategy and identification of initiatives

A structured literature and policy search was conducted between December 2025 and August 2026 to identify potentially relevant national and regional programs, strategies, policy initiatives, and guideline-based approaches addressing asthma and/or allergic diseases. Searches were performed iteratively in PubMed/MEDLINE, the Cochrane Library, and BASE. Scientific database searches were complemented by targeted searches for grey literature and primary program documents using Google and Google Scholar and websites of relevant governmental agencies, professional societies, patient organizations, and national or international health organizations.

Search terms combined disease-related terms (“allergy”, “allergic disease”, “allergology”, “asthma”) with terms describing organized healthcare strategies or programs (“national action plan”, “national program/programme”, “national strategy”, “allergy program/programme”, “asthma program/programme”, “implementation program”, “disease management program”) and, where appropriate, terms related to implementation and evaluation (“implementation”, “evaluation”, “outcome”, “healthcare utilization”, “cost”, “education”, “stakeholder”). The search was conducted in several iterations to identify both potentially relevant initiatives and publications or policy documents associated with individual programs. The detailed search terms used for each source are provided in Supplementary Table S2.

Reference lists of relevant reviews, program reports, guidelines, and identified publications were additionally screened for potentially eligible initiatives and associated implementation or evaluation studies. Only sources available in English or German were considered.

Potentially relevant initiatives were initially assessed against the predefined eligibility criteria by one author and subsequently reviewed by a second author. Disagreements regarding inclusion or classification were resolved by discussion and consensus. No formal risk-of-bias assessment was performed because the evidence base comprised heterogeneous program evaluations, policy documents, and grey literature.

When multiple publications or documents referred to the same initiative, they were grouped at the program level. Primary program documents and publications describing program design and implementation were used to characterize the initiative, while associated evaluation studies were used to extract reported outcomes and follow-up data. Coding was based on information explicitly reported in primary program or strategy documents and associated scientific publications. Program structure, objectives, implementation activities, stakeholder involvement, and evaluation approaches were primarily derived from program or strategy descriptions, whereas reported outcomes were extracted from associated evaluation studies. Information from multiple sources referring to the same initiative was considered jointly at the program level.

Coding was initially performed by one author and subsequently reviewed by a second author. Discrepancies in the assignment of coding categories were discussed and resolved by consensus.

The search and supplementary source assessment identified 13 potentially relevant initiatives. Following application of the eligibility and classification criteria, eight were included in the primary comparative analysis and five were retained as contextual sources. Reasons for exclusion from the primary comparative analysis are provided in Supplementary Table S1.

Supplementary Table S1: Reasons for exclusion of initiatives from primary comparative analysis

**Initiative, Year**	**Classification**	**Reason for exclusion from primary comparative analysis**
UK, 2003	Needs assessment/recommendations	Recommendations for improved allergy services rather than an implemented national program with a defined implementation and evaluation framework
Germany G1, 2007-2012	Historical national policy initiative	Insufficient documentation of a sustained implementation and evaluation framework for systematic comparison across analytical domains
Germany G2, 2014	Professional-society proposal	Call and proposed framework for a future NAP rather than an implemented national program
Germany G3, 2020	Political initiative	Political proposal for coordinated national action rather than an implemented program with evaluable implementation structures or outcomes

India 2026	Clinical guideline/consensus recommendations	Evidence-based clinical recommendations rather than a national health-system implementation program with implementation or outcome data

Data extraction and coding

Relevant information was extracted and systematically organized using a structured coding framework developed to support comparison across the identified initiatives. Extracted information was grouped into six broad categories:

- Action Fields (Prevention, Job Education, Patient Education, Diagnostics, Therapy, Disease Management, Guidelines/Policies, Research, Finance, Reform),
- Tasks (Awareness, Training, Patient Education, Knowledge Assessment, Monitoring/Evaluation, Care Pathways, Multi-disciplinary Care, Research, Guidelines/Laws, Savings, Care Improvements, Tolerance Increase, Reforms),
- Goals (Increase Awareness, Enhance Professional Education, Improve Diagnosis, Achieve Standardized Care, Enhance Disease Control, Boost Self-management, Improve Care Access, Advance Research, Reduce Morbidity, Decrease Costs, Increase the Number of Allergologists, Integrate into Daily Work, Develop Political Strategies, and Establish Monitoring),
- Stakeholders (Patients, Patient Advocacy Groups, Physicians, Nurses, Pharmacists, Societies, Insurances, Education, Research, Government, Industry, Media),
- Evaluation (Population Surveys, Patient Register, Database/Surveillance, Guideline Monitoring, Process Evaluation, Outcome Evaluation), and
- Results (Increased Awareness/Knowledge, Improved Disease Control, Enhanced Quality of Life, Better Diagnostics, Improved Management, Reduced Prevalence, Decreased Occupational Diseases, Lower Hospitalization, Fewer Emergencies, Reduced Costs, Fewer Sick Days, and Development of Education Programs).

Within these categories, individual items were coded as shown in Supplementary Table S3.

Coding was based on information reported in primary program or strategy documents and associated scientific publications. Program structure, objectives, implementation activities, stakeholder involvement, and evaluation approaches were primarily derived from program or strategy descriptions, whereas reported outcomes were extracted from associated evaluation studies. When multiple documents or publications referred to the same initiative, the available information was considered jointly at the program level.

Each item was assigned to one of four coding categories:

- “explicit focus” when the component was directly described as an objective, action, task, organizational element, evaluation measure, or reported outcome of the initiative;

- “indirectly addressed” when the component was not explicitly designated as a program element but was clearly reflected in the described implementation activities or program structure;
- “not mentioned” when no corresponding information was identified in the reviewed sources. “Not mentioned” was interpreted as absence of identifiable information in the reviewed sources and not as evidence that the respective component was absent from the initiative; and
- “not applicable”, when the respective component or outcome was not applicable to the initiative because of its design, scope, or lack of an implementation or evaluation framework.

Coding was initially performed by one author and subsequently reviewed by a second author. Discrepancies in the assignment of coding categories were discussed and resolved by consensus.

Within the “Results” section, coding indicates whether the respective outcome was reported in association with the initiative. It does not indicate the magnitude of change, statistical significance, or causal effectiveness of the program. Quantitative outcome estimates were not compared across initiatives because of heterogeneity in study designs, populations, definitions, and follow-up periods. Directional arrows indicate the direction of the reported change but not its magnitude or statistical significance.

The detailed program-level coding is presented in Supplementary Table S3.

The detailed coding categories in Supplementary Table S3 were grouped iteratively into nine higher-level analytical domains reflecting recurring structural and implementation features across the initiatives. These domains formed the framework for the cross-program comparative synthesis presented in Table 2 of the main manuscript. Reported outcomes were extracted descriptively and were not used to infer the effectiveness of individual program components.

Contextual initiatives not included in the primary comparative analysis

United Kingdom (2003)

In 2003, a Royal College of Physicians Working Party published *Allergy: The Unmet Need*, a needs assessment and set of recommendations aimed at improving allergy services in the United Kingdom [39, 40]. The report identified substantial gaps in specialist allergy care and proposed a coordinated model in which most patients would be managed in primary care, supported by regional specialist allergy centres for patients with more complex conditions. Strengthening specialist capacity and improving access to appropriately trained allergists were identified as important prerequisites for such a model.

The recommendations also emphasized education and training for physicians and nurses, improved allergy knowledge among healthcare professionals, and the development of

multidisciplinary services. Proposed teams included allergists and clinical immunologists as well as professionals from other relevant disciplines, supported by patient organizations and health authorities. Additional priorities included improved public information, strengthened research, and adequate resources for service development and professional training [39, 40]. This initiative was retained as a contextual source because it primarily constituted a needs assessment and set of recommendations for service development rather than an implemented national program with a defined implementation and evaluation framework.

Germany (G1), 2007–2012

In 2007, the German Federal Ministry of Food, Agriculture and Consumer Protection (BMELV) launched the *Action Plan against Allergies* (*Aktionsplan gegen Allergien*) [35, 36]. The initiative aimed to strengthen allergy prevention and reduce the individual and societal burden associated with allergic diseases. Its objectives included improving quality of life and consumer safety, strengthening prevention and early detection, improving public information, and supporting research and evidence-based measures [35, 36].

The Action Plan comprised eight areas of activity: food and consumption; beauty and personal care; clothing and toys; outdoor environments and mobility; monitoring; communication; building and living; and research. Measures included improved allergen labelling, consumer information and counselling, reduction of allergenic exposures, control of highly allergenic plants such as ragweed, support for research, and improved cooperation among policymakers, patient organizations, scientific institutions, and other relevant stakeholders [35, 36]. An online allergy information portal was established as part of the initiative and attracted substantial public interest [41, 42]. The Action Plan ended in 2012. A separate Allergy Information Service (*Allergieinformationsdienst*) was subsequently established in 2016 with federal funding [43, 44]. Further activities related to the Action Plan included recommendations by the German Federal Institute for Risk Assessment concerning exposure to fragrances in public spaces and the need for additional research [45].

The initiative was retained as a contextual source because, despite its broad national scope and multiple areas of action, the available documentation did not provide a sufficiently sustained implementation and evaluation framework for systematic comparison across all analytical domains.

Germany (G2), 2014

In 2014, the *Aktionsforum Allergologie* (AfA), representing German professional societies in allergology, proposed the establishment of a National Action Plan for Allergy (*Nationaler Aktionsplan Allergie*) [10]. The proposal called for coordinated action involving relevant decision-makers and stakeholders and identified several priority areas, including public

awareness, prevention and education, continuing professional education, timely access to appropriate medical care, and prospective evaluation of the proposed action plan.

Suggested measures addressed primary prevention, early recognition and diagnosis, evidence-based treatment, tolerance induction, occupational allergies, severe allergic disease, and healthcare costs. The proposal also emphasized structural improvements in allergy care and the involvement of health policymakers, health insurance organizations, medical professional bodies, and patient organizations [10]. Specific measurable targets and repeated assessments were proposed to evaluate changes in allergy-related knowledge, healthcare utilization, work and school absenteeism, mortality, work incapacity, and direct and indirect costs over time. The proposal was developed in the context of contemporaneous evidence-based recommendations on allergy prevention [46].

The initiative was retained as a contextual source because it represented a professional-society proposal and framework for a future National Action Plan rather than an implemented national program with documented implementation and outcome evaluation.

Germany (G3), 2020

In 2020, a parliamentary initiative called for a coordinated national action program to reduce the individual and societal burden of allergic diseases and improve allergy care in Germany [11]. The proposal emphasized coordinated action across relevant stakeholders and addressed information and education on the development, prevention, treatment, and management of allergic diseases.

Additional areas of proposed action included evidence-based allergy prevention, improved information on avoidable exposure factors, product labelling, air quality, research, and monitoring. The initiative also called for allergy-related information to be integrated across relevant settings in healthcare and education [11].

The initiative was retained as a contextual source because it constituted a political proposal for coordinated national action rather than an implemented program with documented implementation structures and evaluable outcomes.

India (2025/2026)

Allergic diseases represent a substantial health burden in India, while allergy care is affected by limitations in specialist availability, standardized diagnostic approaches, and formal postgraduate training in allergology and clinical immunology [47-49]. Against this background, the Indian Academy of Pediatrics and its Allergy and Applied Immunology Chapter developed evidence-based recommendations for the diagnosis and management of pediatric allergic diseases, including allergic rhinitis, atopic dermatitis, asthma, urticaria, anaphylaxis, and food allergy [50].

The recommendations were developed by a multidisciplinary expert group comprising pediatricians, pulmonologists, otolaryngologists, clinical immunologists, internists, allergists, professional associations, and centres of expertise affiliated with the World Allergy Organization. Development included a systematic review of the literature, structured assessment of the available evidence, and consideration of feasibility, equity, and resource implications in the Indian healthcare context [50]. Recommendations addressed standardized diagnostic approaches, patient and family education, timely referral, evidence-based treatment, appropriate use of diagnostic testing and allergen immunotherapy, professional education, training structures, and policy development. Consensus on recommendations was reached through iterative discussion among the participating experts [50].

The initiative was retained as a contextual source because it primarily represents evidence-based clinical guideline and consensus recommendations rather than an implemented national health-system program with documented implementation and outcome data.

Supplementary Table S2: Search strategy and search terms

The search and supplementary source assessment were conducted between December 2025 and August 2026.

**Source**	**Search terms/ strategy**	**Search purpose**
PubMed/ MEDLINE	"national allergy action plan"<br>"national asthma action plan"<br>allergy AND "national program"<br>asthma AND "national program"<br>allergy AND "national strategy"<br>asthma AND implementation AND program	Identification of programs and associated scientific publications
Cochrane Library	"national allergy action plan"<br>"national asthma action plan"<br>allergy AND "national program"<br>asthma AND "national program"<br>allergy AND "national strategy"<br>asthma AND implementation AND program	Identification of reviews/evaluations and related programs
BASE	"national allergy action plan"<br>"national asthma action plan"	Grey literature and program documents

	allergy AND "national program"<br>asthma AND "national program"<br>allergy AND "national strategy"<br>asthma AND implementation AND program	
Targeted web search	"national allergy action plan"<br>"national asthma action plan"<br>allergy AND "national program"<br>asthma AND "national program"<br>allergy AND "national strategy"<br>asthma AND implementation AND program	Governmental, professional-society and primary program documents
Reference-list screening	References of identified reviews, program reports, guidelines and publications	Additional programs and associated implementation/evaluation publications

Supplementary Table S3: Detailed comparative coding of identified initiatives

Program		NAC	FAsthmaP	FAllergyP	TEAM	UK*	Pro AR	G1*	PCAPP	POLASTMA***	G2*	G3*	IN*	NAS
**Year**		1990	1994-2004	2008-18	1999-2007**	2003	2003-06	2007-12	2003-2006	2008-14	2014	2020	2025 2026	2026
**Population** **N**ational **R**egional		N	N	N	R	N	R	N	R	N	N	N	N	N
**Action Fields**	Prevention	○	●	●	○	○	●	●	○	●	●	●	○	●
	Professional Education	●	●	●	●	● ○	●	○	●	●	●	○	●	●
	Patient Education	●	●	●	●	●	●	●	●	●	●	●	●	●
	Diagnostics	○	●	●	●	●	○	●	○	●	●	○	●	●
	Therapy	○	●	●	●	●	●	○	○	●	●	●	●	●
	Disease Management	●	●	●	●	●	●	○	●	●	○	●	●	●
	Guidelines/ Policies	○	●	●	●	○	○	○	●	○	○	●	●	●
	Research	○	●	◌	◌	○	●	●	○	●	○	●	●	●
	Finance	○	●	●	○	●	○	●	○	●	○	○	○	●
	Reform	○	●	●	●	●	●	○	○	●	●	●	○	●
**Tasks**	Awareness	●	●	●	●	○	●	●	○	●	●	●	●	●
	Training	●	●	●	●	●	●	○	●	●	●	○	●	●

	Patient Education	●	●	●	●	◌	●	●	●	●	●	●	●	●
	Knowledge Assessment	○	●	●	●	●	○	○	●	●	●	○	○	◌
	Monitoring/ Evaluation	●	●	●	●	○	●	●	●	●	●	●	○	●
	Care Pathways	○	●	●	●	●	●	○	●	●	○	○	●	●
	Multi-disciplinary Care	○	●	●	●	●	●	○	○	●	○	○	●	●
	Research	○	●	◌	●	◌	●	●	○	○	○	●	○	●
	Guidelines/ Laws	○	●	●	●	○	○	○	●	●	◌	●	●	●
	Savings	○	●	●	○	○	●	○	○	○	○	○	○	●
	Care Improvement	○	●	●	●	●	●	●	○	●	●	●	●	●
	Tolerance ↑	○	○	●	○	○	○	○	○	○	●	○	○	◌
	Reforms	○	●	●	●	●	◌	○	●	●	○	○	○	●

**Goals**	Awareness ↑	●	●	●	○	○	●	●	●	●	●	●	●	●
	Professional Education ↑	●	●	●	●	●	●	○	○	●	●	○	●	●
	Diagnosis	○	●	●	○	●	○	●	○	●	●	○	●	●
	Standardised Care	●	●	●	●	●	●	○	●	●	○	●	●	●
	Disease Control ↑	●	●	●	●	●	●	●	●	●	●	●	●	●
	Self-management ↑	●	●	●	●	○	◌	○	●	○	○	○	○	●
	Care Access ↑	○	○	○	●	●	●	●	○	◌	●	●	●	●
	Research ↑	○	●	○	○	○	●	●	○	●	○	●	○	●
	Morbidity ↓	●	●	●	●	●	●	●	●	●	●	●	○	◌
	Costs ↓	○	●	●	●	○	●	●	○	●	●	◌	○	●
	Number of Allergologists ↑	○	○	○	○	●	○	○	○	○	○	○	○	●
	Integration into Routine Care	○	●	●	●	○	○	○	○	○	○	○	○	●
	Policy Development	○	○	○	○	○	○	○	○	○	●	●	●	●
	Monitoring	○	●	●	●	○	○	●	○	○	●	●	○	●
**Stakeholder**	Patients	●	●	●	●	○	●	○	●	●	○	○	●	●
	Patient Advocacy	○	●	●	●	●	○	●	○	●	●	●	◌	●
	Physicians	●	●	●	●	●	●	○	●	●	●	●	●	●
	Nurses	○	●	●	●	●	●	○	●	●	○	○	○	●
	Pharmacists	●	●	●	●	●	●	○	○	●	○	○	○	◌
	Societies	●	●	●	●	●	○	○	○	○	●	○	●	●

	Insurances	○	○	●	○	○	○	○	○	●	●	○	○	◌
	Education	○	●	●	●	○	○	○	○	○	○	●	●	●
	Research	○	●	○	●	●	○	○	●	●	○	○	○	●
	Government	○	●	●	●	●	●	●	○	●	●	●	○	●
	Industry	○	●	○	●	○	○	●	○	○	○	○	○	●
	Media	●	●	●	○	○	○	○	○	○	○	○	○	◌
**Evaluation**	Population Surveys	●	●	●	○	NA	○	NA	○	●	●	NA	NA	◌
	Patient Register	○	●	●	○		○		○	○	○			●
	Database/ Surveillance	○	●	●	●		●		○	●	○			●
	Guideline Monitoring	◌	●	◌	●		○		●	●	○			●
	Process Evaluation	●	●	●	●		●		●	●	●			●
	Outcome Evaluation	●	●	●	●		●		●	●	●			●
**Results**	Awareness/ Knowledge	●↑	●↑	●↑	●↑	NA	○	NA	○	○	NA	NA	NA	NA
	Disease Control	○	●↑	●↑	●↑		●↑		●↑	● ○				
	Quality of Life	○	●↑	◌	●↑		●↑		○	○				
	Diagnostics	○	●↑	●↑	○		●↑		●↑	○				
	Management	●↑	●↑	●↑	●↑		●↑		●↑	○				
	Prevalence	●↓	●↓	●↓	●		○		○	○				
	Occupational Diseases	○	●↓	●↓	○		○		○	○				
	Hospitalisation	●↓	●↓	●↓	○		●↓		○	○				

	Emergencies	○	●↓	●↓	○		●↓		●↓	○				
	Costs	○	●↓	●↓	○		●		○	○				
	Sick Days	○	●↓	●	○		●↓		●↓	○				
	Education Programs	○	●	●	●		●		●	●				
**Reference**		[27-29, 33]	[15, 24]	[16, 25, 26]	[17, 18]	[39, 40]	[19, 21, 22, 31, 38]	[35, 36, 41-45]	[30]	[34]	[10]	[11]	[49,50]	[23]

Abbreviations: NAC, National Asthma Campaign; FAsthmaP, Finnish Asthma Programme; FAllergyP, Finnish Allergy Programme; TEAM, Towards Excellence in Asthma Management; ProAR, Program for Control of Asthma and Allergic Rhinitis; PCAPP, Primary Care Asthma Pilot Project; POLASTMA, National Programme of Early Diagnosis and Therapy of Asthma; G1–G3, German initiatives 1–3; IN, Indian initiative; NAS, UK National Allergy Strategy; N, national; R, regional; NA, not applicable.

Coding: ●, explicit focus; ◌, indirectly addressed; ○, not mentioned; NA, not applicable. For reported outcomes in the Results section, ● indicates that the respective outcome was reported in association with the initiative; arrows indicate only the direction of the reported change (↑ increase/improvement where applicable; ↓ decrease), not its magnitude, statistical significance, or causal attribution. *Contextual initiative not included in the primary cross-program comparison. Coding reflects the presence of the respective component or outcome in the reviewed sources and does not imply causality or effectiveness. **The final report describes an eight-year research cycle ending with the second intervention series in 2007; individual preparatory activities had begun earlier. ***Process and outcome evaluation were planned but no corresponding outcome data were identified in the reviewed sources.
